# Comparison of the Proximate Composition and Nutritional Profile of Byproducts and Edible Parts of Five Species of Shrimp

**DOI:** 10.3390/foods10112603

**Published:** 2021-10-27

**Authors:** Zhenyang Liu, Qiumei Liu, Di Zhang, Shuai Wei, Qinxiu Sun, Qiuyu Xia, Wenzheng Shi, Hongwu Ji, Shucheng Liu

**Affiliations:** 1 College of Food Science and Technology, Guangdong Ocean University, Guangdong Provincial Key Laboratory of Aquatic Product Processing and Safety, Guangdong Province Engineering Laboratory for Marine Biological Products, Guangdong Provincial Engineering Technology Research Center of Seafood, Key Laboratory of Advanced Processing of Aquatic Product of Guangdong Higher Education Institution, Zhanjiang 524088, China; liuzhenyang@stu.gdou.edu.cn (Z.L.); 2111903018@stu.gdou.edu.cn (Q.L.); zjs578180838@sina.com (D.Z.); weis@gdou.edu.cn (S.W.); sunqx@gdou.edu.cn (Q.S.); qiuyu@gdou.edu.cn (Q.X.); jihw62318@163.com (H.J.); 2 College of Food Science and Technology, Shanghai Ocean University, Shanghai 201306, China; wzshi@shou.edu.cn; 3 Collaborative Innovation Center of Seafood Deep Processing, Dalian Polytechnic University, Dalian 116034, China

**Keywords:** nutritional component, nutritional value, shrimp

## Abstract

The nutritional components of different parts (meat, head, shell and tail) of *Litopenaeus vannamei* (*L.v*), *Macrobrachium rosenbergii* (*M.r*), *Penaeus monodon* (*P.m*), *Fenneropenaeus chinensis* (*F.c*), and *Penaeus japonicus* (*P.j*) were analyzed and their nutritional values were evaluated. For the five species of shrimp, the meat yield was 37.47–55.94%, and the byproduct yield was 44.06–62.53%. The meat yields of *L.v* and *F.c* were the highest (55.94 and 55.92%, respectively), and the meat yield of *M.r* was the lowest (37.47%). The shrimp contain high amounts of crude protein, and the values of the amino acid score (AAS), chemical score (CS), and essential amino index (EAAI) were greater than or close to 1.00, indicating that shrimp protein had higher nutritional value. The shrimp head was rich in polyunsaturated fatty acids and the ratio of n-6 to n-3 PUFAs was from 0.37 to 1.68, indicating that the shrimp head is rich in n-3 PUFAs and is a good source of n-3 PUFAs. The five species of shrimp were rich in macro- and micro-minerals, especially in shrimp byproducts. The shrimp byproducts were also rich in other bioactive ingredients (astaxanthin), which are also very valuable for developing biological resources. Therefore, shrimp have many nutritional benefits, and their byproducts can also be used to develop natural nutraceuticals, which are considered to be one of the healthiest foods.

## 1. Introduction

Shrimp and shrimp products are among the most commonly consumed seafoods because of their delicacy and nutritional value [1]. In 2020, global shrimp production reached 5.03 million tons and this is expected to grow at a rate of 6.1% over the next few years until 2025 when production is estimated to reach 7.28 million tons [2]. Farmed shrimp contribute 55% of global shrimp production. Globally, shrimp are available in numerous species such as *Litopenaeus vannamei* (*L.v*), *Macrobrachium rosenbergii* (*M.r*), *Penaeus monodon* (*P.m*), *Fenneropenaeus chinensis* (*F.c*), and *Penaeus japonicus* (*P.j*). These varieties are popular in the international market, and many countries are encouraging shrimp production. Asia is the continent with the largest production of farmed shrimp in the world, with the largest production of shrimp farmed in China, followed by Thailand, Indonesia, India, Vietnam and Bangladesh; it has boosted the economies of developing countries, and has generated considerable revenue [3]. However, the global shrimp industry also faces some challenges, such as insufficient utilization of byproducts.

In general, shrimp processing is mainly frozen products, and the products can be divided into three categories: (1) whole shrimp, (2) deheaded shrimp, and (3) peeled shrimp [4]. In the processing of shrimp, byproducts containing the head and shell account for approximately 50–60% of the whole shrimp [5]. Currently, some shrimp byproducts are used as animal feed or aquaculture feed additives, but most of the byproducts are being discarded, causing environmental pollution due to unregulated discharge [6]. In fact, shrimp byproducts contain valuable bioactive components, such as chitin/chitosan, protein/peptides, pigments, enzymes, and lipids, which have been exploited in high value-added products and applied in feed, food, and flavoring [3,7,8,9,10,11]. The maximum recovery of bioactive components from shrimp byproducts is not only conducive to the shrimp processing economy but is also conducive to reducing environmental pollution.

The nutrient/contaminant profile of shrimp is affected by many factors, such as shrimp species, feed, and breeding region. In this study, we focused on the nutritional components of five farmed species shrimp in Zhanjiang, China. Zhanjiang is one of the main shrimp breeding regions in China and has the title of “the capital of shrimp in China”. In this paper, the basic nutritional components of different parts of *L.v*, *M.r*, *P.m*, *F.c*, and *P.j* from Zhanjiang were determined, and their nutritional value was evaluated to provide basic data for shrimp processing and the use of byproducts for value-added products. Meanwhile, this knowledge could be advantageous to help biotechnologists and R&D staff to develop shrimp and byproducts for the sustainable development of the environment.

## 2. Materials and Methods

### 2.1. Materials

**Figure 1:**
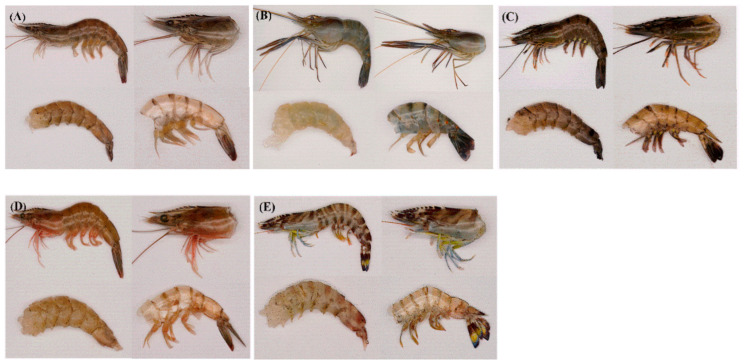
Different parts from five species of shrimp. (**A**) *L.v*, (**B**) *M.r*, (**C**) *P.m*, (**D**) *F.c*, (**E**) *P.j.* For each species of shrimp, from top left to bottom right, the whole shrimp, shrimp head, shrimp meat, shrimp shell and shrimp tail are shown.

Five species of live shrimp (*L.v, M.r, P.m, F.c,* and *P.j*) were purchased from a local seafood market (Dongfeng Seafood Market, Zhanjiang, China), and transported to the laboratory in vivo to ensure the freshness. Five species of shrimp purchased were farmed in summer (from April to June) in Zhanjiang, and 30 shrimp of each species were selected for the experiment. The individual characteristics of shrimp are shown in Table 1. Each species of shrimp was divided into four parts: shrimp head, shrimp shell, shrimp tail, and shrimp meat (Figure 1). The yield of each part of the shrimp was calculated by a weighing method using the following equation, W (g/100 g) = 100×W_1_/W_2_, where W, W_1,_ and W_2_ are the yield, the weight of each separated part (meat, head, shell and tail), and the weight of the whole shrimp, respectively. The samples were stored in a refrigerator at −20 °C until analysis. 

### 2.2. Determination of Proximate Components

The proximate components of shrimp were quantified using the AOAC methods (2006) [12]. Moisture assessment was conducted using oven-drying at 105 °C (AOAC 950.46), crude protein was determined using the micro-Kjeldahl procedure (AOAC 928.08), crude fat was determined using Soxhlet extraction (AOAC 991.36), crude ash was determined using incineration in a muffle furnace at 550 °C (AOAC 920.153), and crude fiber was the organic residue after digestion using H_2_SO_4_ or NaOH.

### 2.3. Determination of Amino Acids

An automatic amino acid analyzer (L-8900; Hitachi, Tokyo, Japan) was used to identify and quantify amino acids [13]. Tryptophan was not determined because it is transformed into ammonium using acidic hydrolysis [14]. 

Approximately 0.5 g of each sample was placed in each tube, adding 10 mL of 6 mol/L hydrochloric acid (Beijing Chemical Works, Beijing, China). The tubes were sealed in a vacuum with enough nitrogen injected to prevent oxidative degradation. At 110 °C, the samples were reacted for 8 h. The reaction product was diluted to 100 mL with distilled water. A volume of 1 mL of reaction product was removed with HCl using vacuum freeze drying. The dried reaction product was dissolved in 2 mL of 0.02 mol/L HCl, and the solution was filtered by a membrane of 0.22 μm pore size (Anpel Laboratory Technologies Inc., Shanghai, China). An aliquot of 20 μL of filtrate was added to the automatic amino acid analyzer, and amino acids were identified by a standard method and quantified by an internal standard method. For the content of amino acid, the data were expressed in g/100 g of sample. The amino acid score (AAS), chemical score (CS), and essential amino acid index (EAAI) were used to evaluate the nutritional value of shrimp protein, the formulas for which are as follows:

(1) $$AAS=\frac{\mathrm{amino}\;\mathrm{acid}\;\mathrm{content}\;\mathrm{in}\;\mathrm{shrimp}\;\mathrm{protein}\;\left(g/100\;g\;\mathrm{protein}\right)}{\mathrm{amino}\;\mathrm{acid}\;\mathrm{content}\;\mathrm{in}\;\mathrm{FAO}/\mathrm{WHO}\;\mathrm{reference}\;\left(g/100\;g\;\mathrm{protein}\right)}$$

(2) $$CS=\frac{\mathrm{amino}\;\mathrm{acid}\;\mathrm{content}\;\mathrm{in}\;\mathrm{shrimp}\;\mathrm{protein}\;\left(g/100\;g\;\mathrm{protein}\right)}{\mathrm{amino}\;\mathrm{acid}\;\mathrm{content}\;\mathrm{in}\;\mathrm{egg}\;\mathrm{protein}\;\left(g/100\;g\;\mathrm{protein}\right)}$$

(3) $$\mathrm{EAAI}=\sqrt[{n}]{\begin{array}{c} \frac{a}{a_{k}}\times \frac{b}{b_{k}}\times \ldots \times \frac{j}{j_{k}} \end{array}}$$

where n is the number of essential amino acids compared, a–j represents the essential amino acid content in shrimp (g/100 g protein), and a_k_–j_k_ indicates the amino acids content of the pattern protein of egg (g/100 g protein). 

### 2.4. Determination of Fatty Acids

According to the method of Wu et al. [15], total lipids were extracted from the samples via chloroform–methanol solution (2:1, *v*/*v*) (Beijing Chemical Works, Beijing, China). The lipids were saponified using a potassium hydroxide-methanol (Beijing Chemical Works, Beijing, China), and transformed to methyl esters using 12.5 *w*/*v* sulfuric acid–methanol (Beijing Chemical Works, Beijing, China). Fatty acid methyl esters (FAMEs) were extracted with n-hexane (Beijing Chemical Works, Beijing, China), which were analyzed using a GCMS-TQ8050NX (Shimadzu, Tokyo, Japan) with a DB-23 (60 m × 0.25 mm × 0.25 µm, Agilent Technologies, Santa Clara, CA, USA). Helium (1.0 mL/min) was used as the carrier gas, and the split ratio was 1:5. The initial temperature of the column was 120 °C for 5 min. The column temperature increased to 240 °C at 4 °C per min and was maintained at 240 °C for 10 min. The column temperature increased to 250 °C at 5 °C per min and was maintained at 250 °C for 25 min. The injector temperature was 280 °C and the ion source temperature was 250 °C. The injection volume was 1 μL. FAMEs were identified by comparing the retention times with those of a mixture of fatty methyl ester standards (Supelco 37 Component FAME Mix; Supelco Inc., Bellefonte, PA, USA). The results were expressed as g/100 g of total fatty acids identified. The retention times for standard FAMEs (Supelco 37 Component FAME Mix; Supelco Inc., Bellefonte, PA, USA) were used to compare and identify FAMEs in samples. Data were represented using g/100 g of total fatty acids identified.

### 2.5. Determination of Minerals

The mineral and heavy metal were determined according to the Lorenzo et al. [16] method using an inductively coupled plasma emission spectrometer (ICAP7400; Thermo Electron, Massachusetts, MA, USA). Approximately 4 g of sample was placed in a PTFE tube, and 12 mL of concentrated nitric acid (68%) (Beijing Chemical Works, Beijing, China) was added. The digestion was carried out until the solution was colorless. After cooling, the solution was transferred to a 50 mL volumetric flask and was diluted to a fixed volume with double-deionized water, while a blank experiment was performed. 

### 2.6. Determination of Astaxanthin

According to the method of Roy et al. [17], extraction of astaxanthin was performed. An amount of 200 mg of sample was placed in a 50 mL centrifuge tube. Then, 5 mL solvent of dichloromethane: methanol (1:3, *v*/*v*) (Beijing Chemical Works, Beijing, China) was added. The mixture was treated in an oscillator (SHY-2, Putian Technologies, Changzhou, Suzhou, China) for 3 h and then centrifuged at 5000 r/min for 15 min at 4 °C. A collection of the supernatant, and 5 mL solvent of dichloromethane: methanol (1:3, *v*/*v*) was added to the precipitate again. The above procedure was repeated three times. The extracts were collected and an equal amount of petroleum ether (Beijing Chemical Works, Beijing, China) was added (boiling point 40–60 °C). After shaking, the separated petroleum ether layer was purged with an MGS-2200H nitrogen purging instrument (EYELLA company, Tokyo, Japan) for 30 min to remove the organic solvent and obtain pure astaxanthin. The dried astaxanthin was dissolved in 5 mL of n-hexane, and then the solution was filtered using a 0.45 μm membrane filter to remove particulate residues. 

The extracts with astaxanthin were determined using HPLC (e2695, Waters, Milford, MA, USA) fitted with a C18 column (4.6 mm × 250 mm × 5 µm, Agilent Technologies, Santa Clara, CA, USA). The mobile phase was methanol and ultrapure water with a flow rate of 1 mL/min. The column temperature was kept at 35 °C. The detection wavelength was 480 nm. The injection volume was 10 µL.

### 2.7. Statistical Analysis

All experiments were repeated three times and experimental data were represented using the mean ± standard deviation. One-way analysis of variance (ANOVA) and Tukey HSD multiple comparisons were performed using JMP10.0 software (SAS, Cary, NC, USA) to analyze significant differences (*p* < 0.05).

## 3. Results

### 3.1. Yield

The meat yield of shrimp is the main technical and economic index of shrimp processing enterprises. As shown in Table 1 and Table 2, the mass of five species varied from 16.00 ± 1.46 to 40.81 ± 3.09 g and the meat yield of five species of shrimp was 37.47–55.94%. The meat yields of *L.v*, *F.c* and *P.j* were significantly higher than those of *P.m* and *M.r* (*p* < 0.05). However, the mass of *P.m* was the highest. The meat yield of *M.r* was the lowest. The meat yield differences may be related to biological characteristics as different shrimp species, even *L.v*, *F.c*, *P.j*, and *M.r*, showed a similar size or mass [18]. 

Shrimp heads, shrimp shells and shrimp tails were the byproducts of shrimp processing, the yields of which were 33.63–53.09%, 7.44–7.74% and 1.73–2.82%, respectively. The total byproduct yield of the five species of shrimp was 44.06–62.53%. Generally, 45–60% of whole shrimp was converted into byproducts (heads, shells and tails) during processing, which differs between species and processing methods [18,19]. These byproducts from shrimp processing are considered an abundant source of potential animal proteins and bioactive components, such as chitin, peptides, oil, astaxanthin, minerals, and flavor compounds. Many reports are available about the preparation of bioactive peptides [20], the extraction of shrimp oil and astaxanthin [10,19,21], the extraction of chitin and preparation of chitosan [22,23], and the development of flavoring [24] from shrimp processing byproducts, some of which have been industrialized. Therefore, shrimp meat and shrimp processing byproducts have a high nutritional and economic value.

### 3.2. Proximate Components

The proximate components of the five species of shrimp are presented in Table 3. From Table 3, the crude protein content in shrimp meat was the highest (12.33–15.09%), followed by the shells and tails and the heads. The highest crude protein content in shrimp meat was found in *L.v* (15.09%), followed by *P.j* (14.60%), *F.c* (13.18%), *P.m* (13.29%) and *M.r* (12.33%). Wu et al. [25] found 17.70–18.71% crude protein in four species of shrimp. The high content of crude protein in shrimp meat is one of the reasons why shrimp is high-quality seafood.

No significant difference (*p* > 0.05) was found in the crude protein content of the shell, tail, and head for the same species of shrimp. The crude protein in byproducts consists of 70–80% myogenic fibronectin and 20–30% sarcoplasmic protein, which gives seafood protein a higher nutritional value [26]. Compared to the other four species of shrimp, the content of crude protein in byproducts from *L.v* was the lowest. 

For the five species of shrimp, a significant difference (*p* < 0.05) was found in the content of crude fat from the same part. For the same species of shrimp, a significant difference (*p* < 0.05) was also found in the content of crude fat from the different parts. The content of crude fat (2.17–6.88%) in shrimp heads was highest compared to shrimp meat and shells and tails. In crustaceans, the hepatopancreas is considered the main organ of metabolism and is also the storehouse of lipids, including triglycerides and phospholipids [10]. 

The ash content reflects the content of inorganic compounds in biological samples to a certain extent. From Table 3, the ash content (8.18–13.45%) in the shells and tails from the five species of shrimp was the highest (*p* < 0.05), followed by the heads (4.11–7.30%) and meat (1.33–2.07%). This result indicated that shrimp was rich in mineral elements.

The crude fiber in shrimp is mainly chitin (poly β-(1-4) *N*-acetyl-*D*-glucosamine), which mainly exists in shrimp shell and is the second most profuse natural polymer on Earth after cellulose [27]. Significant differences (*p* < 0.05) were found in the crude fiber content of different parts for the same species of shrimp. The crude fiber contents (7.86–10.78%) in shrimp shells and tails were the highest, followed by the heads (2.38–3.99%). The crude fiber content reported by Ali et al. [19] reached 18.2% in shrimp byproducts. *Kritchenkov* et al. [28] reported that chitin derivatives exhibit good antibacterial properties as membrane coatings. Chitosan obtained from crude fiber is not only biodegradable and biocompatible but also has positive biological properties; these properties have been used make coating films that have been successfully applied to preserve seafood with longer shelf life and better quality by antimicrobial and antioxidant effects [22,23].

The above analysis shows that there were significant differences (*p* < 0.05) in the same component in the same part from different species of shrimp and significant differences (*p* < 0.05) in the same component in different parts of the same species of shrimp. These differences were related to several biotic and abiotic factors, including the species, age, and nutritional status [29]. 

### 3.3. Amino Acid Composition

Generally, the quality of protein in food mainly depends on the protein content, essential amino acid content and proportion, and digestibility [30]. In our study, we evaluated the nutritional value of shrimp protein based on essential amino acid content and proportion according to the recommendations of WHO/FAO.

From Table 4, sixteen kinds of amino acids were detected in the five species of shrimp, among which seven kinds of essential amino acids were detected (tryptophan was not detected because it was destroyed by acid hydrolysis). The content of amino acids in different species of shrimp or different parts of shrimp exhibited significant differences (*p* < 0.05). Most of the amino acid contents and the total amino acid content in shrimp meat were higher in shrimp meat than in shrimp heads, shells and tails (*p* < 0.05), which was consistent with the protein content in Table 3.

According to the essential amino acid model recommended by the FAO/WHO, the ratio of EAAs/TAAs should be greater than or equal to 0.40 [31], and the ratio of NEAAs/EAAs should be greater than or equal to 0.60. From Table 4, it can be determined that the ratio of EAAs/TAAs in shrimp was 0.35~0.40, which is very close to 0.40; the ratio of EAAs/NEAAs in shrimp was 0.52–0.66, which is very close to 0.60. Many approaches are used in evaluating protein quality, and the common approaches are AAS, CS, and EAAI. AAS is the proportion of amino acids in 1 g of sample protein compared to the FAO/WHO recommended amino acid requirement. CS is the proportion of amino acids in 1 g of sample protein to that in 1 g of egg white protein. EAAI is a geometric means to calculate the overall comparison between all essential amino acids in a target protein and those in the reference protein. In general, the value of AAS or CS is above 1, which indicates that the nutritional value of a target protein is high. If the value of EAAI is above 0.95, a target protein is of high quality; if the value of EAAI is between 0.85 and 0.95, a target protein is better; if the value of EAAI is between 0.75 and 0.86, a target protein is available; if the value of EAAI is below 0.75, a target protein is not suitable [32]. The AAS, CS, and EAAI values of shrimp protein are shown in Table 5. All the AAS and EAAI values of protein in different parts of the five species of shrimp were above 1.00. Although the CS values of partial essential amino acids were below 1.00, they were very close to 1.00. These results indicated that the protein from shrimp meat or byproducts was of high quality, which was consistent with the EAAs/TAAs and EAAs/NEAAs results.

The contents of glycine, alanine, glutamic acid, and aspartic acid are closely related to the umami of seafood. Table 4 shows that the contents of four umami amino acids in shrimp meat or byproducts were high, and the ratio of DAAs/TAAs was 0.36–0.41, indicating that shrimp processing byproducts are important materials for developing shrimp seasoning.

In summary, shrimp are an important resource of dietary amino acids for humans. Shrimp contains high-content protein, and the proportions of amino acids are balanced relative to human requirements. Therefore, shrimp protein has a high nutritional value.

### 3.4. Fatty Acid Profile

The profile and content of fatty acids in food are important indexes for evaluating the nutritional value of lipids. Saturated fatty acids (SFAs) play an important role in the energy metabolism of shrimp. However, excessive saturated fatty acid intake in humans is not conducive to human health and may increase the risk of cardiovascular disease. Oleic acid in monounsaturated fatty acids (MUFAs) is also the main energy source in the process of shrimp growth and metabolism. Polyunsaturated fatty acids (PUFAs) are the bioactive substances with unique physiological functions, and their nutritional value is positively correlated with the degree of unsaturated fatty acids. 

The crude fat content in shrimp meat and shrimp shell was low, and the fatty acid profile was not analyzed. The crude fat content of shrimp heads was high, and the fatty acid profile of shrimp heads is summarized in Table 6. SFAs (61.54–73.16 g/100 g) were the primary fatty acids, followed by PUFAs (16.26–30.33 g/100 g) and MUFAs (6.05–15.83 g/100 g). The SFAs were dominated by myristic acid (C14:0), palmitic acid (C16:0), and stearic acid (C18:0), with relative contents of 3.27–10.88 g/100 g, 16.90–32.76 g/100 g, and 11.5–25.93 g/100 g, respectively. The MUFAs were mainly oleic acid (C18:1) and palmitoleic acid (C16:1), with relative contents ranging from 3.17 to 12.66 g/100 g and 2.08 to 5.38 g/100 g, respectively. From the perspective of nutrition, MUFAs have many effects on human health. Recent evidence has shown that MUFAs can reduce the risk of cardiovascular diseases and other inflammation-related diseases, but the effects of different MUFAs are different [33]. PUFAs were mainly EPA (C20:5n-3) and DHA (C22:6n-3), with relative contents from 3.08 to 10.29 g/100 g and 4.33 to 6.44 g/100 g, respectively. PUFAs are considered to have a lowering effect on cholesterol and hypertriglyceridemia compared to SFAs, but different unsaturated fatty acids have different properties [34]. Studies have shown that DHA has a preventive and therapeutic effect on neurological disorders, DHA also has a good health effect on the brain and vision development in infants and adolescents, and EPA has a good preventive and therapeutic effect on cardiovascular system diseases [35]. The DHA/EPA values in shrimp heads were from 0.53 to 0.89 except for *F.c*, indicating that the lipids in the other four species of shrimp heads are cardiovascular protective and can be used to develop nutraceuticals for the protection of the cardiovascular system. The DHA/EPA value in the *F.c* head was 1.59, indicating that lipids from *F.c* heads can be used to develop educational and brain-healthy products and promote vision development [35].

The WHO/FAO recommends that the ratio of n-6 to n-3 PUFAs intake should be 4–6 in the diet, while the current ratio of n-6 to n-3 PUFAs intake is as high as 10 in the Chinese diet, which indicates a severe lack of n-3 PUFAs in the Chinese diet [31]. The n-6 to n-3 PUFAs ratio in shrimp heads was 0.37–1.68, which is less than the WHO/FAO recommended standard, indicating that shrimp heads were rich in n-3 PUFAs and were a good source of n-3 PUFAs.

### 3.5. Minerals

The mineral component of shrimp is shown in Table 7. The content of minerals in different species of shrimp or different parts of shrimp exhibited significant differences (*p* < 0.05). Shrimp byproducts are rich in calcium (Ca) and magnesium (Mg) in the ranges of 12,350–43,300 µg/g and 591–1900 µg/g, respectively, which makes them a good high-calcium food source. All parts of shrimp were rich in potassium (K), sodium (Na) and phosphorus (P), with contents ranging from 982 to 4030 µg/g, 899.5–3280 µg/g and 252.5–493 µg/g, respectively. They maintain the acid-base balance of body fluids and cellular metabolism in the human body [36]. Na and K were effective in promoting energy metabolism and are essential for the maintenance of nerves, cell membrane permeability and normal cell function [37]. In addition, some studies have shown that the ideal ratio of Na to K is less than 1.5, and a ratio that is too high is not conducive to the balance of Na and K in the human body [36]. In this study, the ratio of Na to K in shrimp was mostly close to or below 1.5 in shrimp, indicating that the proportion of Na and K in shrimp is suitable for the human body. For microelements, iron (Fe), zinc (Zn) and copper (Cu) were present in all parts of the shrimp, especially in shrimp byproducts, with contents ranging from 2.72 to 36.65 µg/g, 5.11 to 16.30 µg/g and 3.21 to 24.85 µg/g, respectively. Fe is mainly found as ferritin in the liver, spleen, and bone marrow, and hemoglobin in human blood is an iron complex that has the function of fixing and transporting oxygen. Zn promotes protein synthesis in the brain and helps to develop and improve the nervous system. Cu plays various physiological and biochemical roles of great importance in the maintenance of metabolism and the cardiovascular system in living organisms [36,38]. The interactions among Fe, Zn, and Cu are antagonistic, and this antagonism usually occurs when the ratio of Zn to Cu is above 10 and the ratio of Zn to Fe is above 1 [39]. The ratio of Zn to Cu was less than 10 in all parts of shrimp except for *P.j* meat. The ratio of Zn to Fe was greater than 1 in shrimp meat, and less than 1 in shrimp byproducts. The results indicated that the ratios of Zn to Cu and Zn to Fe were more desirable in shrimp byproducts than in shrimp meat.

Shrimp heads are susceptible to the enrichment of heavy metals, which results in reduced nutritional value. The possible reason is that aquatic animals can accumulate heavy metals from different sources, including sediments, airborne dust and discharge of wastewater [40]. It has been reported that the hepatopancreas is a metal storage and detoxification organ in crustaceans, and the direct contact of the gills with water accelerates the enrichment of heavy metals [41].

According to the FAO/WHO (2016) [41], the limiting values for Pb, Cd, As and Hg are 0.50 µg/g. Table 6 shows that the cadmium (Cd) content in the shrimp head was significantly higher (*p* < 0.05) than that in the shrimp shell, shrimp tail and shrimp meat, with 0.10–0.75 µg/g. Pb was detected in low amounts (0.15 µg/g) in the heads of *L.v*. Arsenic (As) was detected in shrimp meat and shrimp byproducts at a range of 0.21–3.75 µg/g, probably due to the high concentration of As in farmed waters [39]. In this experiment, only total arsenic content was detected, and organic arsenic and inorganic arsenic compounds were not distinguished. Inorganic arsenic is toxic, while organic arsenic has little or no toxicity [42]. The excessive content of total arsenic in shrimp does not mean that it is toxic. Therefore, it is necessary to further analyze the contents of inorganic arsenic and organic arsenic compounds. Hg was not detected. Cd and Pb in bones are difficult to decompose, have a long half-life, and can cause acute or chronic poisoning when taken in excess. The contents of Pb and Hg in shrimp were lower than the limiting values, while the content of Cd in the head of *M.r* was slightly higher than the limiting values; the content of As in the shrimp meat and part of the byproducts of the five shrimp species was slightly higher than the limiting values.

### 3.6. Astaxanthin

**Figure 2:**
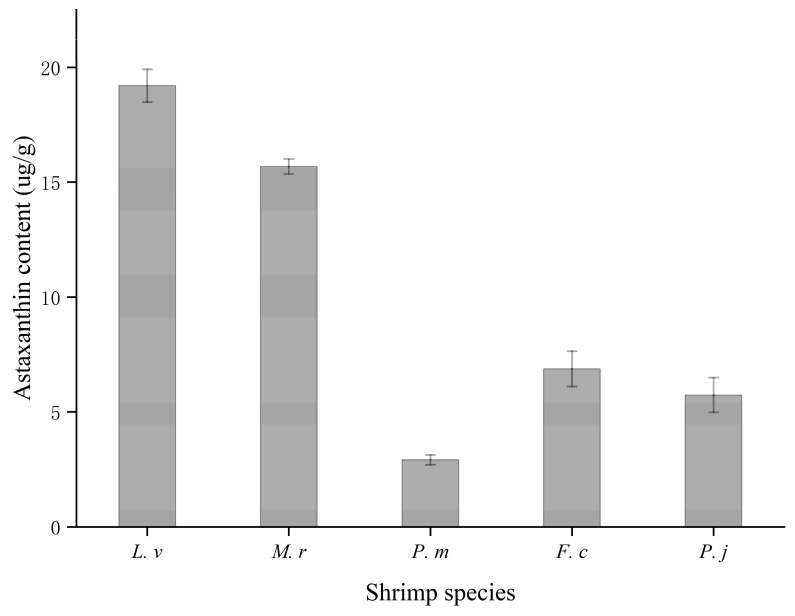
Content of astaxanthin extracted from five species of shrimp byproducts (head, shell and tail).

Astaxanthin (3,3-dihydroxy-β, β-carotene-4,4-dione) is the primary carotenoid present in shrimp processing byproducts and can be found in its free or esterified form. The content of astaxanthin in shrimp processing byproducts is shown in Figure 2. From Figure 2 it can be determined that the highest level (19.20 µg/g) of astaxanthin was found in byproducts of *L.v*, followed by *M.r* (15.68 µg/g) and the lowest level was found in *P.m* (2.91 µg/g). This result was similar to that of Ali et al. [19] using supercritical CO_2_ extraction. The low level of astaxanthin in P. m may be due to a lack of astaxanthin in the diet. Astaxanthin can scavenge free radicals and has antioxidant activity 500-fold greater than that of tocopherol (vitamin E). Pan et al. [43] obtained a significant increase in the antioxidant activity of oil containing astaxanthin. Some authors have reported the use of astaxanthin extracted from shrimp processing byproducts as an additive in fish feed and nutritional products [44,45]. Astaxanthin is not only used as a dietary supplement for humans and animals but also has anti-diabetic, anticancer, immune-boosting, cardiovascular disease prevention, and fertility-enhancing effects [46].

## 4. Conclusions

The nutritional components in the five species of shrimp were analyzed and their nutritional values were evaluated. The meat yield and the byproduct yield of five species of shrimp were 37.47–55.94% and 44.06–62.53%, respectively. The meat yields of *L.v* and *F.c* were the highest (55.94 and 55.92%, respectively), and the meat yield of *M.r* was the lowest (37.47%). The content of crude protein in shrimp meat was the highest (12.33–15.09%), the essential amino acids were rich, and the values of AAS, CS, and EAAI were more than or close to 1.00, indicating that shrimp protein had higher nutritional value. The shrimp head was rich in polyunsaturated fatty acids and the ratio of n-6 to n-3 PUFAs was 0.37–1.68, indicating that shrimp head is rich in n-3 PUFAs and is a good source of n-3 PUFAs. The five species of shrimp were rich in macro- and micro-minerals, especially in shrimp byproducts. The ratio of Na to K was close to or below 1.5, indicating that the ratio of Na to K is appropriate for human requirements. The ratio of Zn to Cu and Zn to Fe indicates that their content is more desirable in shrimp byproducts than in shrimp meat. The shrimp byproducts were also rich in other bioactive ingredients (astaxanthin), which are also very valuable for developing biological resources.

Although shrimp byproducts are rich in nutritional and bioactive components, the content of heavy metals, especially cadmium, exceeds the limiting value, which also poses some challenges for the utilization of shrimp byproducts. Therefore, when using shrimp byproducts to develop high value-added products, it is necessary to consider removing excess heavy metals.

## Figures and Tables

**Table 1:** Length and mass of five species of shrimp.

Species	*L.v*	*M.r*	*P.m*	*F.c*	*P.j*
Length/cm	13.46 ± 0.42	13.60 ± 0.57	17.44 ± 0.39	14.33 ± 0.45	13.42 ± 0.54
Mass/g	16.00 ± 1.46	24.52 ± 2.70	40.81 ± 3.09	21.78 ± 2.54	16.98 ± 2.24

Note: *L.v*, *M.r*, *P.m*, *F.c*, and *P.j* are abbreviations of *Litopenaeus vannamei*, *Macrobrachium rosenbergii*, *Penaeus monodon*, *Fenneropenaeus chinensis*, and *Penaeus japonicus*, respectively.

**Table 2:** Yield of shrimp meat and byproducts.

Species	Yield (g/100 g)			
	Meat	Head	Shell	Tail
*L.v*	55.94 ± 2.46 a	33.63 ± 1.65 d	7.61 ± 0.89 a	2.82 ± 0.41 a
*M.r*	37.47 ± 1.22 d	53.09 ± 1.42 a	7.71 ± 0.86 a	1.73 ± 0.21 c
*P.m*	47.92 ± 1.68 c	41.92 ± 2.45 b	7.44 ± 0.62 a	2.72 ± 0.32 a
*F.c*	55.92 ± 0.87 a	34.26 ± 0.94 d	7.57 ± 0.50 a	2.25 ± 0.16 b
*P.j*	52.14 ± 2.03 b	37.91 ± 2.04 c	7.74 ± 0.25 a	2.21 ± 0.31 b

Note: The difference in the same part yield from different species of shrimp was analyzed and different letters indicate significant differences (*p* < 0.05).

**Table 3:** Proximate components of shrimp meat and byproducts (g/100 g sample).

Composition	Species	Head	Shell and Tail	Meat
Crude protein	*L.v*	6.56 ± 0.01 Bd	7.98 ± 0.09 Bc	15.09 ± 0.02 Aa
	*M.r*	8.81 ± 0.04 Bc	11.30 ± 0.21 Ba	12.33 ± 0.03 Ad
	*P.m*	9.14 ± 0.17 Bb	11.05 ± 0.08 Ba	13.29 ± 0.18 Ac
	*F.c*	8.75 ± 0.02 Bc	10.43 ± 0.17 Bb	13.18 ± 0.03 Ac
	*P.j*	9.97 ± 0.10 Ba	10.60 ± 0.21 Bb	14.60 ± 0.07 Ab
Crude fat	*L.v*	3.78 ± 0.02 Ac	0.66 ± 0.05 Bb	0.70 ± 0.02 Ba
	*M.r*	6.88 ± 0.05 Aa	0.78 ± 0.03 Ba	0.80 ± 0.03 Ba
	*P.m*	3.04 ± 0.07 Ad	0.51 ± 0.02 Bc	0.48 ± 0.02 Bb
	*F.c*	3.88 ± 0.03 Ab	0.52 ± 0.04 Bc	0.68 ± 0.00 Ba
	*P.j*	2.17 ± 0.03 Ae	0.41 ± 0.01 Cd	0.73 ± 0.01 Ba
Ash	*L.v*	4.11 ± 0.14 Bd	8.57 ± 0.08 Ac	1.87 ± 0.02 Cb
	*M.r*	6.05 ± 0.15 Bb	13.31 ± 0.15 Aa	1.33 ± 0.00 Ce
	*P.m*	5.58 ± 0.16 Bc	10.72 ± 0.03 Ab	1.56 ± 0.02 Cd
	*F.c*	4.18 ± 0.06 Bd	8.18 ± 0.08 Ad	1.74 ± 0.02 Cc
	*P.j*	7.30 ± 0.07 Ba	13.45 ± 0.12 Aa	2.07 ± 0.03 Ca
Crude fiber	*L.v*	2.93 ± 0.03 Bc	7.91 ± 0.09 Ad	0.38 ± 0.04 Ca
	*M.r*	3.99 ± 0.04 Ba	10.78 ± 0.01 Aa	0.29 ± 0.00 Cab
	*P.m*	3.56 ± 0.06 Bb	9.74 ± 0.06 Ac	0.30 ± 0.05 Cab
	*F.c*	2.38 ± 0.05 Bd	7.86 ± 0.10 Ad	0.28 ± 0.03 Cb
	*P.j*	3.10 ± 0.13 Bc	10.25 ± 0.03 Ab	0.35 ± 0.03 Cab
Moisture	*L.v*	72.97 ± 0.25 Ab	66.07 ± 0.42 Bb	73.76 ± 0.08 Ac
	*M.r*	68.12 ± 0.91 Bd	58.63 ± 0.47 Cd	77.57 ± 0.13 Aa
	*P.m*	72.22 ± 0.51 Bbc	62.90 ± 0.68 Cc	75.55 ± 0.23 Ab
	*F.c*	75.36 ± 0.61 Aa	68.10 ± 0.68 Ba	75.70 ± 0.15 Ab
	*P.j*	70.55 ± 0.32 Bc	61.28 ± 0.25 Cc	72.97 ± 0.03 Ad

Note: The difference in the same component in the same part from different species of shrimp was analyzed and different lowercase letters indicate significant differences (*p* < 0.05); the differences in the same component in different parts of the same species of shrimp were analyzed and different uppercase letters indicate significant differences (*p* < 0.05).

**Table 4:** Amino acid contents of shrimp meat and byproducts (g/100 g sample).

Amino Acids	Head					Shell + Tail					Meat				
	*L.v*	*M.r*	*P.m*	*F.c*	*P.j*	*L.v*	*M.r*	*P.m*	*F.c*	*P.j*	*L.v*	*M.r*	*P.m*	*F.c*	*P.j*
Lys	0.86 ± 0.01 bc	0.85 ± 0.02 bcd	0.83 ± 0.04 cd	0.88 ± 0.00 b	0.85 ± 0.02 d	0.74 ± 0.01 e	0.86 ± 0.00 bc	0.66 ± 0.03 g	0.71 ± 0.00 ef	0.68 ± 0.0 fg	1.69 ± 0.01 a	1.71 ± 0.01 a	1.70 ± 0.01 a	1.71 ± 0.01 a	1.71 ± 0.00 a
Phe	0.60 ± 0.01 i	0.68 ± 0.01 g	0.60 ± 0.01 i	0.62 ± 0.00 h	0.68 ± 0.01 hi	0.73 ± 0.01 ef	0.87 ± 0.01 a	0.69 ± 0.00 g	0.74 ± 0.01 e	0.72 ± 0.00 f	0.80 ± 0.02 d	0.81 ± 0.00 cd	0.82 ± 0.01 bcd	0.82 ± 0.00 bc	0.83 ± 0.01 b
Leu	0.82 ± 0.00 ef	0.82 ± 0.02 ef	0.83 ± 0.03 ef	0.84 ± 0.00 de	0.82 ± 0.02 a	0.76 ± 0.00 g	0.87 ± 0.00 d	0.70 ± 0.03 h	0.74 ± 0.00 gh	0.73 ± 0.00 gh	1.49 ± 0.01 c	1.51 ± 0.01 bc	1.53 ± 0.01 ab	1.52 ± 0.00 bc	1.56 ± 0.01 a
IIe	0.56 ± 0.00 g	0.61 ± 0.01 ef	0.58 ± 0.01 g	0.58 ± 0.01 g	0.61 ± 0.01 g	0.61 ± 0.01 ef	0.76 ± 0.00 d	0.61 ± 0.01 ef	0.60 ± 0.00 f	0.62 ± 0.00 e	0.82 ± 0.02 c	0.87 ± 0.01 a	0.81 ± 0.02 c	0.82 ± 0.01 c	0.84 ± 0.01 b
Val	0.61 ± 0.00 g	0.65 ± 0.01 f	0.62 ± 0.01 g	0.63 ± 0.01 f	0.65 ± 0.01 g	0.73 ± 0.02 d	0.83 ± 0.00 b	0.69 ± 0.01 e	0.75 ± 0.00 c	0.72 ± 0.00 d	0.83 ± 0.00 b	0.88 ± 0.00 a	0.84 ± 0.01 b	0.85 ± 0.00 b	0.88 ± 0.00 a
Thr	0.54 ± 0.00 gh	0.55 ± 0.00 gh	0.52 ± 0.02 i	0.53 ± 0.00 hi	0.55 ± 0.00 hi	0.59 ± 0.01 d	0.67 ± 0.00 c	0.55 ± 0.01 fg	0.57 ± 0.00 ef	0.58 ± 0.00 de	0.76 ± 0.00 b	0.75 ± 0.00 b	0.76 ± 0.01 b	0.76 ± 0.00 b	0.79 ± 0.00 a
Met	0.50 ± 0.00 fg	0.56 ± 0.02 cde	0.54 ± 0.01 def	0.57 ± 0.00 bcde	0.56 ± 0.02 g	0.57 ± 0.14 bcd	0.61 ± 0.00 ab	0.62 ± 0.02 a	0.52 ± 0.01 ef	0.55 ± 0.00 cde	0.55 ± 0.00 cde	0.54 ± 0.00 def	0.57 ± 0.00 bcd	0.55 ± 0.00 cde	0.59 ± 0.00 abc
Arg	0.93 ± 0.00 e	0.77 ± 0.00 g	0.79 ± 0.03 g	0.95 ± 0.00 e	0.77 ± 0.00 g	0.94 ± 0.00 e	0.95 ± 0.00 e	0.83 ± 0.02 f	0.92 ± 0.01 e	0.94 ± 0.00 e	2.01 ± 0.01 b	1.78 ± 0.01 d	1.77 ± 0.01 d	1.89 ± 0.01 c	2.12 ± 0.01 a
His	0.29 ± 0.00 j	0.40 ± 0.00 d	0.30 ± 0.01 ij	0.31 ± 0.00 hi	0.40 ± 0.00 fg	0.32 ± 0.00 gh	0.45 ± 0.00 b	0.31 ± 0.00 gh	0.34 ± 0.00 e	0.33 ± 0.00 ef	0.40 ± 0.00 d	0.47 ± 0.01 a	0.40 ± 0.00 d	0.42 ± 0.00 c	0.45 ± 0.00 b
Tyr	0.48 ± 0.01 hi	0.55 ± 0.00 f	0.48 ± 0.00 hi	0.51 ± 0.01 g	0.55 ± 0.00 i	0.53 ± 0.00 g	0.65 ± 0.00 d	0.49 ± 0.01 h	0.57 ± 0.01 e	0.48 ± 0.00 hi	0.69 ± 0.00 c	0.71 ± 0.01 bc	0.72 ± 0.01 ab	0.70 ± 0.00 c	0.72 ± 0.01 a
Ala#	0.83 ± 0.00 j	0.74 ± 0.00 k	0.86 ± 0.03 ij	0.86 ± 0.00 i	0.74 ± 0.00 k	1.00 ± 0.02 f	0.93 ± 0.00 g	1.05 ± 0.01 e	1.05 ± 0.00 e	0.90 ± 0.00 h	1.14 ± 0.00 cd	1.12 ± 0.01 d	1.37 ± 0.01 a	1.25 ± 0.00 b	1.17 ± 0.01 c
Gly#	0.99 ± 0.04 e	0.67 ± 0.01 h	0.92 ± 0.01 f	0.98 ± 0.01 e	0.67 ± 0.01 f	1.20 ± 0.01 cd	0.92 ± 0.00 f	1.16 ± 0.02 d	1.22 ± 0.00 c	1.18 ± 0.00 cd	1.38 ± 0.00 b	0.86 ± 0.00 g	1.52 ± 0.01 a	1.36 ± 0.00 b	1.56 ± 0.01 a
Pro	0.68 ± 0.00 f	0.58 ± 0.00 h	0.63 ± 0.00 g	0.73 ± 0.00 e	0.58 ± 0.00 h	0.80 ± 0.02 d	0.83 ± 0.01 c	0.88 ± 0.01 b	0.97 ± 0.01 a	0.79 ± 0.00 d	0.79 ± 0.00 d	0.65 ± 0.00 g	0.86 ± 0.01 b	0.97 ± 0.01 a	0.81 ± 0.01 d
Glu#	1.73 ± 0.00 ghi	1.56 ± 0.03 j	1.74 ± 0.05 fgh	1.76 ± 0.00 fg	1.56 ± 0.03 i	1.74 ± 0.02 fgh	1.83 ± 0.01 e	1.69 ± 0.04 hi	1.79 ± 0.01 ef	1.69 ± 0.00 hi	3.11 ± 0.00 c	3.00 ± 0.01 d	3.24 ± 0.03ab	3.19 ± 0.01b	3.27 ± 0.02a
Ser	0.52 ± 0.01 hi	0.57 ± 0.01 g	0.52 ± 0.01 hi	0.53 ± 0.00 h	0.57 ± 0.01 i	0.67 ± 0.00 e	0.79 ± 0.00 a	0.65 ± 0.01 f	0.68 ± 0.00 e	0.64 ± 0.00 f	0.74 ± 0.00 cd	0.76 ± 0.00 bc	0.73 ± 0.01 d	0.74 ± 0.00 d	0.76 ± 0.00 b
Asp#	1.20 ± 0.01 i	1.27 ± 0.00 f	1.21 ± 0.03 hi	1.25 ± 0.00 fg	1.27 ± 0.00 i	1.31 ± 0.01 e	1.46 ± 0.00 c	1.23 ± 0.03 gh	1.35 ± 0.01 d	1.31 ± 0.00 e	1.98 ± 0.00 b	2.06 ± 0.00 a	2.00 ± 0.02 b	2.00 ± 0.01 b	2.04 ± 0.01 a
TAA	12.14	11.83	11.97	12.53	11.83	13.24	14.23	12.81	13.52	12.86	19.18	18.48	19.64	19.55	20.10
EAA	4.49	4.72	4.52	4.65	4.72	4.73	5.47	4.52	4.63	4.60	6.94	7.07	7.03	7.03	7.20
NEAA	7.65	7.11	7.45	7.88	7.11	8.51	8.76	8.29	8.89	8.26	12.24	11.41	12.61	12.52	12.90
DAA	4.75	4.24	4.73	4.85	4.24	5.25	5.14	5.13	5.41	5.08	7.61	7.04	8.13	7.80	8.04
EAA/NEAA	0.59	0.66	0.61	0.59	0.66	0.56	0.62	0.55	0.52	0.56	0.57	0.62	0.56	0.56	0.56
EAA/TAA	0.37	0.40	0.38	0.37	0.40	0.36	0.38	0.35	0.35	0.36	0.36	0.38	0.36	0.36	0.36
DAA/TAA	0.39	0.36	0.40	0.39	0.36	0.40	0.36	0.40	0.40	0.40	0.40	0.38	0.41	0.40	0.40

Note: Values in the same row that do not share the same superscript letter are significantly different (*p* < 0.05). # and DAA refer to the delicious amino acid, EAA refers to the essential amino acid, TAA refers to the total amino acid, NEAA refers to the non-essential amino acid.

**Table 5:** AAS, CS, and EAAI of protein from shrimp meat and byproducts.

Part	Species	Amino Acid	Thr	Val	Met	Ile	Leu	Phe + Tyr	Lys	EAAI
Head	*L.v*	*AAS*	2.06	1.87	2.18	2.13	1.78	3.12	3.41	1.61
		*CS*	1.83	1.47	1.29	1.71	1.41	1.73	1.96	
	*M.r*	*AAS*	1.56	1.49	1.82	1.73	1.32	2.64	2.51	1.27
		*CS*	1.39	1.12	1.08	1.38	1.10	1.47	1.44	
	*P.m*	*AAS*	1.42	1.37	1.69	1.59	1.29	2.24	2.36	1.17
		*CS*	1.26	1.03	1.00	1.27	1.07	1.24	1.36	
	*F.c*	*AAS*	1.51	1.45	1.86	1.66	1.36	2.45	2.62	1.26
		*CS*	1.35	1.09	1.10	1.33	1.13	1.36	1.50	
	*P.j*	*AAS*	1.33	1.23	1.62	1.43	1.14	2.03	2.12	1.56
		*CS*	1.23	0.99	0.96	1.22	0.97	1.28	1.27	
Shell and Tail	*L.v*	*AAS*	1.85	1.84	2.04	1.91	1.35	2.99	2.41	1.41
		*CS*	1.64	1.39	1.21	1.53	1.12	1.66	1.38	
	*M.r*	*AAS*	1.48	1.48	1.54	1.68	1.09	2.55	1.98	1.15
		*CS*	1.32	1.11	0.91	1.35	0.91	1.42	1.14	
	*P.m*	*AAS*	1.24	1.26	1.60	1.38	0.90	2.02	1.56	1.00
		*CS*	1.11	0.95	0.95	1.10	0.75	1.12	0.89	
	*F.c*	*AAS*	1.37	1.45	1.42	1.44	1.01	2.38	1.77	1.05
		*CS*	1.21	1.09	0.85	1.15	0.83	1.32	1.02	
	*P.j*	*AAS*	1.37	1.37	1.48	1.46	0.98	2.14	1.67	1.03
		*CS*	1.22	1.03	0.88	1.17	0.81	1.19	0.96	
Meat	*L.v*	*AAS*	1.26	1.11	1.04	1.36	1.40	1.87	2.92	1.03
		*CS*	1.12	0.83	0.62	1.09	1.16	1.04	1.67	
	*M.r*	*AAS*	1.52	1.44	1.25	1.76	1.74	2.33	3.61	1.29
		*CS*	1.35	1.08	0.74	1.41	1.44	1.30	2.07	
	*P.m*	*AAS*	1.43	1.27	1.23	1.52	1.64	2.19	3.33	1.20
		*CS*	1.27	0.96	0.73	1.22	1.35	1.22	2.06	
	*F.c*	*AAS*	1.90	1.30	1.19	1.56	1.64	2.18	3.38	1.20
		*CS*	1.28	0.98	0.71	1.24	1.36	1.21	1.94	
	*P.j*	*AAS*	1.35	1.22	1.15	1.44	1.52	2.01	3.05	1.11
		*CS*	1.20	0.91	0.68	1.15	1.26	1.12	1.75	

**Table 6:** Fatty acid profile of shrimp heads (g/100 g of total fatty acids).

Fatty Acid	*L.v*	*M.r*	*P.m*	*F.c*	*P.j*
C12:0	0.05 ± 0.00 b	1.05 ± 0.00 a	0.12 ± 0.00 b	0.05 ± 0.00 c	0.19 ± 0.00 b
C13:0	0.03 ± 0.00 b	0.59 ± 0.00 a	0.04 ± 0.00 b	0.06 ± 0.00 c	0.08 ± 0.00 b
C14:0	3.27 ± 0.00 c	10.88 ± 0.01 a	9.28 ± 0.01 a	4.49 ± 0.00 c	6.54 ± 0.00 b
C15:0	1.74 ± 0.00 c	4.33 ± 0.00 a	1.77 ± 0.00 c	2.40 ± 0.00 c	3.11 ± 0.00 b
C16:0	32.76 ± 0.01 a	18.20 ± 0.02 ab	16.90 ± 0.29 ab	31.86 ± 0.01 a	30.04 ± 0.02 a
C17:0	2.74 ± 0.00 b	4.94 ± 0.00 a	2.80 ± 0.00 b	2.56 ± 0.00 b	4.48 ± 0.00 a
C18:0	16.90 ± 0.01 c	11.50 ± 0.03 d	25.93 ± 0.02 a	14.94 ± 0.01 c	23.53 ± 0.01 b
C20:0	0.87 ± 0.00 b	2.36 ± 0.00 a	0.92 ± 0.00 b	1.08 ± 0.00 b	1.02 ± 0.00 b
C21:0	0.32 ± 0.00 c	1.13 ± 0.00 a	0.18 ± 0.00 b	0.51 ± 0.00 b	0.20 ± 0.00 b
C22:0	1.00 ± 0.00 b	1.99 ± 0.00 a	0.82 ± 0.00 b	1.44 ± 0.00 b	0.59 ± 0.00 b
C23:0	2.33 ± 0.00 b	3.22 ± 0.00 a	2.03 ± 0.00 b	1.13 ± 0.00 c	3.10 ± 0.00 a
C24:0	0.62 ± 0.00 a	1.35 ± 0.01 a	0.75 ± 0.00 a	0.71 ± 0.00 a	0.23 ± 0.00 b
C16:1	2.08 ± 0.00 c	5.38 ± 0.00 a	3.41 ± 0.00 b	2.30 ± 0.00 c	3.63 ± 0.00 b
C17:1	0.11 ± 0.00 b	0.58 ± 0.00 a	0.19 ± 0.00 b	0.25 ± 0.00 b	0.51 ± 0.00 b
C18:1	10.48 ± 0.00 b	3.17 ± 0.00 d	3.29 ± 0.00 d	12.66 ± 0.01 a	5.71 ± 0.00 c
C20:1	0.81 ± 0.00 a	0.25 ± 0.00 b	0.71 ± 0.01 b	1.20 ± 0.00 a	0.61 ± 0.00 a
C22:1	0.06 ± 0.00 a	0.11 ± 0.00 a	0.11 ± 0.00 a	0.19 ± 0.00 a	0.04 ± 0.00 a
C24:1	0.19 ± 0.00 a	0.14 ± 0.00 a	0.33 ± 0.00 a	0.23 ± 0.00 a	0.06 ± 0.00 a
C18:2n-6	10.34 ± 0.00 b	0.01 ± 0.00 d	11.04 ± 0.00 ab	10.83 ± 0.00 a	0.82 ± 0.00 c
C18:3n-3	0.02 ± 0.00 a	0.26 ± 0.00 a	0.04 ± 0.00 a	0.01 ± 0.00 a	0.03 ± 0.00 a
C18:3n-6	0.91 ± 0.00 b	3.29 ± 0.01 a	0.74 ± 0.00 b	0.74 ± 0.00 b	0.35 ± 0.00 c
C20:2n-6	0.88 ± 0.00 b	2.77 ± 0.03 a	0.65 ± 0.00 b	0.95 ± 0.00 b	0.57 ± 0.00 b
C20:3n-3	0.12 ± 0.00 a	0.28 ± 0.00 a	0.12 ± 0.00 a	0.07 ± 0.00 a	0.12 ± 0.00 a
C20:3n-6	0.16 ± 0.00 b	2.20 ± 0.00 a	0.12 ± 0.00 b	0.14 ± 0.00 c	0.09 ± 0.00 b
C20:4n-6	0.33 ± 0.00 c	1.99 ± 0.00 a	0.13 ± 0.00 c	0.69 ± 0.00 b	0.04 ± 0.00 c
C20:5n-3(EPA)	5.15 ± 0.00 c	10.29 ± 0.00 a	9.85 ± 0.00 b	3.08 ± 0.00 d	7.25 ± 0.00 b
C22:2n-6	0.09 ± 0.00 a	0.18 ± 0.00 a	0.10 ± 0.00 a	0.05 ± 0.00 a	0.04 ± 0.00 a
C22:4n-6	0.80 ± 0.00 c	1.59 ± 0.00 b	0.92 ± 0.00 d	0.23 ± 0.00 e	1.60 ± 0.00 a
C22:5n-3	0.11 ± 0.00 b	0.23 ± 0.00 b	0.05 ± 0.00 b	0.04 ± 0.00 b	0.90 ± 0.00 a
C22:5n-6	0.12 ± 0.00 a	0.14 ± 0.00 a	0.13 ± 0.00 a	0.07 ± 0.00 a	0.12 ± 0.00 a
C22:6n-3(DHA)	4.57 ± 0.00 a	5.47 ± 0.00 a	6.44 ± 0.06 a	4.90 ± 0.00 a	4.33 ± 0.00 a
SFAs	62.65	61.54	63.63	62.35	73.16
MUFAs	13.72	9.63	6.05	15.83	10.58
PUFAs	23.6	28.7	30.33	21.8	16.26
PUFAs/SFAs	0.38	0.47	0.48	0.35	0.22
EPA+DHA	9.72	15.76	16.29	7.98	11.58
DHA/EPA	0.89	0.53	0.65	1.59	0.60
n-6/n-3	1.36	0.75	0.83	1.68	0.37

Note: Values in the same row that do not share the same superscript letter are significantly different (*p* < 0.05).

**Table 7:** Mineral composition of shrimp meat and byproducts (µg/g sample).

Parts	Species	K	Na	Ca	Mg	Fe	Zn	P	Cu	Pb	As	Cd	Na: K	Zn: Cu	Zn: Fe
Head	*L.v*	2030.0 ± 70.7 e	2920.0 ± 63.6 cd	12,350.0 ± 777.8 h	964.0 ± 65.1 e	34.4 ± 1.1 b	11.3 ± 0.2 bc	344.5 ± 3.5 f	14.5 ± 0.5 b	0.2 ± 0.1	1.3 ± 0.0 e	ND	1.4	0.9	0.3
	*M.r*	1510.0 ± 42.4 g	1510.0 ± 7.1 i	23,050.0 ± 353.6 e	591.0 ± 2.8 fg	34.1 ± 1.8 b	16.3 ± 1.0 a	351.0 ± 5.7 f	24.9 ± 1.2 a	ND	1.2 ± 0.1 fg	0.8±0.1 a	1.0	0.7	0.5
	*P.m*	1910.0 ± 14.1 e	3280.0 ± 63.6 a	15,600.0 ± 636.4 g	699.0 ± 25.5 f	23.1 ± 0.8 c	12.0 ± 0.6 b	309.5 ± 10.6 h	12.8 ± 0.2 c	ND	0.4 ± 0.1 i	0.1±0.1 d	1.7	0.9	0.5
	*F.c*	2060.0 ± 35.4 e	2860.0 ± 35.4 d	13,550.0 ± 353.6 h	1110.0 ± 56.6 d	22.8 ± 0.2 c	10.2 ± 0.1 d	325.0 ± 8.5 g	24.3 ± 0.1 a	ND	2.0 ± 0.1 b	0.2±0.1 c	1.4	0.4	0.5
	*P.j*	1990.0 ± 21.2 e	3010.0 ± 14.1 c	19,350.0 ± 495.0 f	1180.0 ± 49.5 d	12.8 ± 0.3 d	11.1 ± 0.3 c	393.0 ± 7.1 d	11.0 ± 0.3 d	ND	0.9 ± 0.1 h	0.3±0.1 b	1.5	1.0	0.9
Shell and tail	*L.v*	1730.0 ± 99.0 f	2680.0 ± 56.6 e	23,300.0 ± 282.8 e	1440.0 ± 99.0 c	7.5 ± 0.1 e	6.5 ± 0.2 gh	493.0 ± 1.4 a	4.8 ± 0.1 g	ND	1.2 ± 0.1 f	ND	1.6	1.4	0.9
	*M.r*	982.0 ± 11.3 h	1050.0 ± 14.1 j	43,300.0 ± 1555.6 a	1210.0 ± 77.8 d	36.7 ± 2.2 a	5.4 ± 0.3 ij	461.5 ± 0.7 b	10.3 ± 0.3 de	ND	0.8 ± 0.1 h	ND	1.1	0.5	0.2
	*P.m*	1018.5 ± 44.6 h	3130.0 ± 42.4 b	30,200.0 ± 0.0 c	1750.0 ± 35.4 b	7.3 ± 0.1 e	7.1 ± 0.2 fg	349.0 ± 5.7 f	10.0 ± 0.4 e	ND	0.2 ± 0.1 j	ND	3.1	0.7	1.0
	*F.c*	1640.0 ± 21.2 fg	2440.0 ± 106.1 g	27,750.0 ± 70.7 d	1810.0 ± 106.1 ab	8.2 ± 0.4 e	5.1 ± 0.3 j	418.5 ± 7.8 c	8.2 ± 0.4 f	ND	1.6 ± 0.1 d	ND	1.5	0.6	0.6
	*P.j*	1550.0 ± 28.3 g	2570.0 ± 35.4 f	33,600.0 ± 1979.9 b	1900.0 ± 106.1 a	2.7 ± 0.2 h	6.0 ± 0.1 hi	484.0 ± 11.3 a	3.2 ± 0.1 h	ND	0.4 ± 0.2 i	ND	1.7	1.9	2.2
Meat	*L.v*	3730.0 ± 155.6 c	1710.0 ± 77.8 h	286.0 ± 4.2 i	426.5 ± 13.4 h	3.2 ± 0.2 gh	8.6 ± 0.3 e	288.5 ± 2.1 i	3.7 ± 0.1 h	ND	1.1 ± 0.1 g	ND	0.5	2.3	2.7
	*M.r*	2810.0 ± 70.7 d	899.5 ± 29.0 k	269.0 ± 14.1 i	257.5 ± 5.0 i	3.0 ± 0.1 h	7.5 ± 0.1 f	252.5 ± 2.1 j	3.9 ± 0.1 h	ND	1.1 ± 0.1 g	ND	0.3	1.9	2.5
	*P.m*	3820.0 ± 148.5 bc	1690.0 ± 49.5 h	229.0 ± 2.8 i	394.5 ± 10.6 h	5.4 ± 0.2 f	9.5 ± 0.2 a	312.5 ± 3.5 gh	3.4 ± 0.1 h	ND	1.1 ± 0.1 g	ND	0.4	2.8	1.8
	*F.c*	3970.0 ± 113.1 ab	1400.0 ± 42.4 i	352.0 ± 7.1 i	507.0 ± 15.6 gh	4.9 ± 0.1 fg	7.0 ± 0.3 fg	313.5 ± 6.4 gh	2.2 ± 0.1 i	ND	1.7 ± 0.2 c	ND	0.4	3.2	1.4
	*P.j*	4030.0 ± 42.4 a	1 420.0 ± 21.2 i	227.0 ± 12.7 i	426.5 ± 29.0 h	3.4 ± 0.1 gh	8.5 ± 0.1 e	378.5 ± 6.4 e	2.3 ± 0.1 i	ND	3.8 ± 0.1 a	ND	0.4	3.6	2.5

Note: Values in the same column that do not share the same superscript letter are significantly different (*p* < 0.05). ND indicates no detection.

## Data Availability

The datasets generated for this study are available on request to the corresponding author.
